# A Case of Chronic Dysentery Successfully Treated by Large Doses of the Vitrum Antimonii Ceratum

**Published:** 1784

**Authors:** 


IV. *A Caſe of chronic dyſentery ſucceſsfully treated by large doſes of the Vitrum Antimonii ceratum. Communicated to Dr.* Simmons *by* Nicholas Chavaſſe, *Member of the Corporation of Surgeons of London, and Surgeon at Walſall, in Staffordſhire.*

THE efficacy of the vitrum antimonii ceratum has been atteſted by ſeveral medical practitioners, who have experienced its utility in cafes of Dyfentery, which had obftinately refifted the remedies ufually employed in this painful and loathfome difeafe; and I feel much pleafure in being able to add my teftimony in favour of a medicine which, if judicioufly employed, is, I am perfuaded, equal to the cure of every dyfenteric complaint that is remediable by art.

In a variety of cafes of this fort I have adminiftered it with fuccefs; but in none has the relief it produced been fo extraordinary as in the following inftance, which I take the liberty of communicating to you, to be inferted, if you think proper, in the London Medical Journal.

### C A S E.

H. Smith, aged 55, had for more than feven years been afflicted with conftant and excruciating pains in his back and bowels, accompanied with tenefmus, which was for the moft part relieved by a difcharge of blood, always to the amount of half a pint, and oftentimes more, in the fpace of twenty-four hours. He had taken a variety of medicines, which had been recommended to him by different pracpractitioners, both in London and in the country; and, according to his own account of his caſe, had not been unmindful of trying ſeveral noſtrums which had been recommended to him by different friends. Nothing he had tried, however, had moderated his complaint except ſome opiate medicines; and when the ſoothing effects of theſe ceaſed, he was ſure to be tortured by additional pain, and debilitated by an increaſed diſcharge of blood. When I firſt ſaw him, he complained of hectic heat, intenſe thirſt, inceſſant pain in his back and bowels, flatulency, dyſpnœa, and loſs of appetite. He made but little water, his belly was tenſe, and his legs much affected with anaſarcous ſwelling. Under ſuch a train of formidable ſymptoms, I could not entertain very ſanguine hopes of ſucceſs, eſpecially when I conſidered the ſeverity and long continuance of his complaints, his age, and his unhealthy calling, which was that of an engineer to a coal mine. I began with directing him to take ten grains of the vitrum antimonii ceratum twice a day. In this doſe it excited very little nauſea, and no increaſed diſcharge by the bowels; the quantity was therefore gradually augmented to twenty grains, when my patient viſibly began to mend, and at the end of twenty-ſeven days the diſcharge was entirely ſuppreſſed. His bowels now became regular, and, if we may judge from the appearance of his ſtools, we may venture to aſſert, that the diameter of the rectum had been much diminiſhed by the ſeverity of his complaint. After the ſuppreſſion of the diſcharge, all his other ſymptoms gradually diſappeared, and in leſs than two months he was reſtored to his former good health and cheerful ſpirits, and remains ſo to this day, which is more than half a year ſince he firſt applied to me. While he was taking the medicine, I directed him to drink a mixture compoſed of equal parts of milk and lime water, which I have often found ſerviceable in reſtoring a due tone to the bowels when weakened by dyſenteric purging.

In addition to the above caſe, I beg to obſerve, that having, during my reſidence in the Weſt Indies, had frequent opportunities of experiencing the ſalutary powers of the vitrum antimonii ceratum in caſes of dyſentery, which had reſiſted the remedies commonly employed in that diſeaſe, I truſt I ſhall be excuſed for venturing to recommend a more frequent trial of this medicine to the ſurgeons both of the army army and navy, who, as well as myself, must often have had the melancholy opportunity of witnessing the fatality of this disease in our West-India islands, where it sweeps away, with unrelenting fury, so many of our soldiers and sailors.

